# Treatment of Metastatic Cancer by Conferring Immunogenicity to the Apoptotic Bodies of the Primary Tumor

**DOI:** 10.34133/cancomm.0001

**Published:** 2026-01-23

**Authors:** So-Jung Kim, Hae-Bin Park, Eun-Koung An, Dayoung Ryu, Da Young Kim, Daeun Lim, Wei Zhang, Xiaoyan Zhang, Jianqing Xu, Peter Chang-Whan Lee, Jun-O Jin

**Affiliations:** ^1^ Department of Microbiology, Brain Korea 21 Project, University of Ulsan College of Medicine, ASAN Medical Center, Seoul, South Korea.; ^2^ Department of Biochemistry and Molecular Biology, Brain Korea 21 Project, University of Ulsan College of Medicine, ASAN Medical Center, Seoul, South Korea.; ^3^ Shanghai Public Health Clinical Center, Fudan University, Shanghai, P.R. China.; ^4^ Laboratory for Immunotherapy, Clinical Center for Biotherapy, Zhongshan Hospital, Fudan University, Shanghai, P.R. China.

The cure rate of cancer is increasing with the development of new treatment methods, including surgical treatments and immunotherapy [1]. Nevertheless, treatment of metastatic cancer remains challenging [2]. Immunotherapy is thought to suppress metastatic cancer, as it promotes specific killing of tumor cells via recognition of cancer antigens [3]. This antigen-specific immunity involving helper T cells and cytotoxic T lymphocyte activation is mediated by antigen presentation and stimulation of dendritic cells (DCs) [4]. Nonetheless, the strategies aimed at treating metastatic cancer by inducing the activation of DCs have not achieved substantial results [5]. Notably, inducing apoptosis in tumor cells has also been explored as a potential therapeutic strategy [6]. Apoptotic bodies (ABs), which are small membrane-bound vesicles released by dying cells during apoptosis, have been investigated as drug delivery vehicles to specifically target and destroy tumors [7]. However, by inducing transforming growth factor-β and interleukin-10, ABs could inhibit macrophage activation, thereby suppressing immune responses to antigens and promoting immune tolerance [8]. Consequently, the therapeutic application of ABs in tumor immunotherapy remains limited due to their potential to promote immune tolerance. Nonetheless, ABs derived from tumor cells contain abundant tumor antigens [9]. If sufficient immune activity can be elicited, these ABs may serve as a potential therapeutic approach for metastatic cancer. Therefore, we hypothesized that generating ABs using tumors extracted from a bilateral tumor model that mimics metastatic/residual disease and enhancing their immunogenicity could provide an effective strategy for treating metastatic cancer (Fig. 1A).

**Fig. 1.**
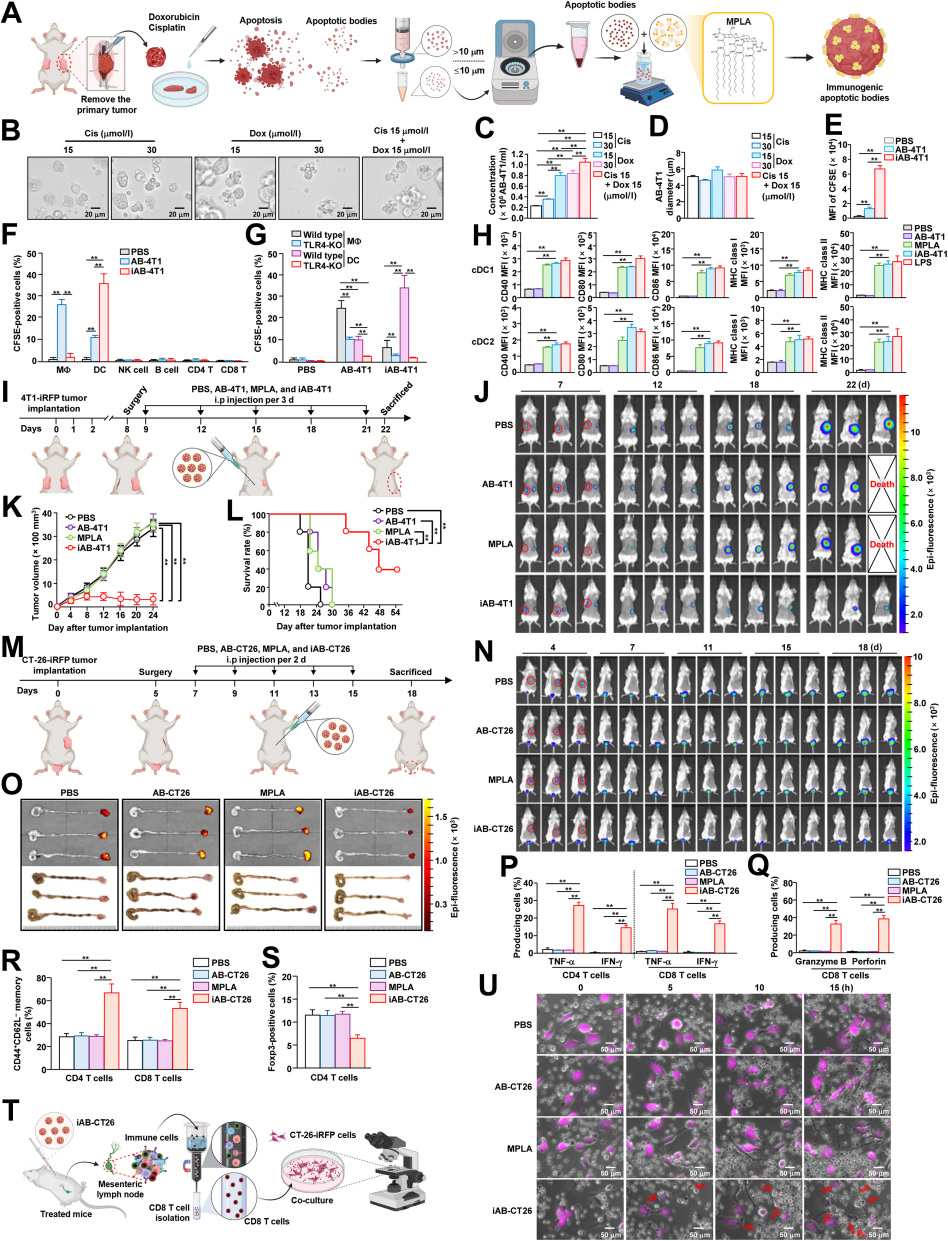
Treatment of metastatic cancer with iABs. (A) Schematic illustration of iAB production. (B) Microscope images of apoptotic 4T1-iRFP tumor cells treated with Cis and Dox for 24 h. (C) Concentration of AB-4T1 following different concentrations and combination of Cis and Dox (*n* = 6 samples, 2-way ANOVA, mean ± SEM, **P < 0.01). (D) AB-4T1 sizes were analyzed using a Luna automated cell counter (*n* = 6 samples, 2-way ANOVA, mean ± SEM). (E) BMDCs were incubated with CFSE-labeled AB-4T1 or iAB-4T1. At 3 h after incubation, the cells were observed under a fluorescence microscope. MFI of CFSE in BMDCs (*n* = 6 samples, 2-way ANOVA, mean ± SEM, **P < 0.01). (F) Detection of CFSE-labeled AB-4T1 or iAB-4T1 taken up by the indicated immune cells was detected performed by flow cytometry 2 h after injection to BALB/c mice (*n* = 6 mice, 2-way ANOVA, mean ± SEM, **P < 0.01). (G) CFSE-labeled AB-4T1 and iAB-4T1 were injected into wild-type and TLR4-KO mice, and 2 h after injection, the mean percentage of CFSE-labeled AB-4T1 and iAB-4T1 taken up in splenic DCs and macrophages was analyzed using flow cytometry (*n* = 6 mice, 2-way ANOVA, mean ± SEM, **P < 0.01). (H) Surface activation marker (CD40, CD80, and CD86) and MHC molecule levels in cDC1 (upper panel) and cDC2 (lower panel) 18 h after injection of PBS, AB-4T1, MPLA (40 μg), iAB-4T1, and LPS. (I) 4T1-iRFP cells were administered into both the left and right fat pads to induce breast cancer. Eight days after tumor administration, when the tumor size reached 100 mm^3^, the right-sided tumor was removed, and iAB-4T1 was produced as shown in panel (A). iAB-4T1 was administered 5 times at 3-d intervals to mice with residual left-sided 4T1-iRFP tumors as shown in the schematic treatment schedule. (J) Fluorescence-based time-dependent tumor growth of 4T1-iRFP during injection of PBS, AB-4T1, MPLA (40 μg), and iAB-4T1. (K) 4T1-iRFP tumor growth curves during treatment with PBS, AB-4T1, MPLA (40 μg), and iAB-4T1 (*n* = 6 mice, significance was determined by the log-rank test, mean ± SEM, **P < 0.01). (L) Survival rate of 4T1-iRFP tumor-bearing mice after treatment with PBS, AB-4T1, MPLA (40 μg), and iAB-4T1 (*n* = 5 mice, significance was determined by the log-rank test, mean ± SEM, **P < 0.01). (M) CT-26-iRFP cells were administered into the rectal area and the left flank. Five days after tumor administration, when the size of the left flank tumor reached around 100 mm^3^, the tumor was excised, and iAB-CT26 was generated. PBS, AB-CT26, MPLA (40 μg), and iAB-CT26 were administered at 2-d intervals starting 7 d after tumor administration, and tumor growth was observed. Schematic schedule for treatment with iAB-CT26. (N) Time-dependent iRFP expression in tumors administered intrarectally during treatment with PBS, AB-CT26, MPLA (40 μg), and iAB-CT26. (O) The iRFP fluorescence expression (upper panel) and tumor mass (lower panel) in the rectal area 18 d after tumor injection are shown. (P) Induction of tumor antigen-specific T cell immunity by iAB-CT26 was analyzed. Eighteen days after CT-26-iRFP cell administration into the rectum, the mice were euthanized, and the mLNs were harvested. mLN cells were stimulated with CT-26-iRFP cell membrane proteins for 15 h. Mean intracellular TNF-α- and IFN-γ-producing cells in mLN CD4 (left panel) and CD8 T cells (right panel, *n* = 6 mice, 2-way ANOVA, mean ± SEM, **P < 0.01). (Q) Mean granzyme B- and perforin-producing cells in CD8 T cells (*n* = 6 mice, 2-way ANOVA, mean ± SEM, **P < 0.01). (R) Mean percentage of CD62L^−^CD44^+^ memory cells in CD4 and CD8 T cells (*n* = 6 mice, 2-way ANOVA, mean ± SEM, **P < 0.01). (S) Mean percentage of Foxp3-positive CD4 T cells in mLNs (*n* = 6 mice, 2-way ANOVA, mean ± SEM, **P < 0.01). (T) Schematic illustration showing the experimental design for evaluating the aggressiveness of cytotoxic T lymphocytes against CT-26-iRFP cells. (U) Time-dependent images from time-lapse video. Purple-colored cells indicate CT-26-iRFP cells, and small cells are CD8 T cells. The red arrow indicates CT-26-iRFP under attack by CTLs. Abbreviations: AB, apoptotic body; ANOVA, analysis of variance; BMDCs, bone-marrow-derived dendritic cells; CFSE, carboxyfluorescein succinimidyl ester; Cis, cisplatin; CD, cluster of differentiation; cDC1, conventional type 1 DC; cDC2, conventional type 2 DC; DC, dendritic cell; CD4 T, CD4 T cell; CD8 T, CD8 T cell; Dox, doxorubicin; Foxp3, forkhead box P3; iAB, immunogenic apoptotic body; i.p., intraperitoneal; iRFP, infrared fluorescent protein; IFN-γ, interferon-γ; KO, knockout; LPS, lipopolysaccharide; MΦ, macrophage; MHC, major histocompatibility complex; MFI, mean fluorescence intensity; mLNs, mesenteric lymph nodes; MPLA, monophosphoryl lipid A; NK cell, natural killer cell; PBS, phosphate-buffered saline; SEM, standard error of the mean; TLR4, Toll-like receptor 4; TNF-α, tumor necrosis factor-α.

To that end, tumors from 4T1 cells expressing infrared fluorescent protein (iRFP), approximately 100 mm^3^ in size, were excised from tumor-bearing mice and processed into single-cell suspensions (Supplementary Materials and Table S1). The 4T1-iRFP tumor cells were then subjected to cisplatin (Cis) and/or doxorubicin (Dox), leading to apoptosis (Fig. S1A and B) and the production of AB (AB-4T1) within 24 h (Fig. 1B). Compared with each agent alone, AB-4T1 production was more efficient when a combination of Cis and Dox was used (Fig. 1C and Table S2). The harvested AB-4T1 was approximately 5 μm in diameter (Fig. 1D). To confer immunogenicity to AB-4T1, monophosphoryl lipid A (MPLA), a detoxified lipopolysaccharide derivative and Toll-like receptor 4 (TLR4) agonist [10], was selected as an immune adjuvant. Lipid insertion into AB-4T1 to produce immunogenic apoptotic bodies (iABs) was assessed using DSPE–PEG2000–fluorescein isothiocyanate, revealing a concentration-dependent increase in insertion, with fluorescence rising stepwise up to 50 μg (Fig. S1C and D). Importantly, MPLA insertion did not induce marked changes in AB size, morphology, or protein composition over the 10-d observation period (Fig. S1E to G).

We then verified DC targeting, showing that iAB-4T1 was effectively phagocytosed by bone-marrow-derived DCs (Fig. 1E and Fig. S2A and B). To assess in vivo uptake, immune cells from the spleen were isolated and analyzed (Fig. S2C). While AB-4T1 was efficiently internalized by F4/80^+^ macrophages, iAB-4T1 exhibited a higher uptake efficiency by DCs (Fig. 1F and Fig. S2D). As previously reported [8], AB-4T1 administered in vivo was predominantly phagocytosed by macrophages, contributing to their clearance and potential immune suppression. Notably, iAB-4T1 uptake by DCs was significantly reduced in TLR4-knockout mice, indicating that this process occurred in a TLR4-dependent manner (Fig. 1G).

To conduct experiments under conditions similar to those in patients with metastatic cancer or residual disease, 4T1-iRFP tumors were implanted on both sides of the mammary gland; the tumors on one side were surgically removed, and iAB-4T1 was generated. Of note, iAB-4T1 containing 40 μg of MPLA markedly enhanced activation marker (CD40, CD80, and CD86) expressions in conventional DC 1 (cDC1) and cDC2 cells within the spleen and tumor-draining lymph nodes, with no additional effect at higher doses. Compared to MPLA alone, iAB-4T1 showed a splenic DC activation efficiency of 97.2% ± 1.2% (Fig. 1H and Figs. S3 and S4).

Then, we evaluated the therapeutic effect of iAB-4T1 in our in vivo tumor model. Eight days after 4T1-iRFP tumor injection on the left side of the mammary gland, iAB-4T1 was administered to the mice 5 times at 3-d intervals (Fig. 1I). Tumor growth was suppressed in iAB-4T1-treated mice compared to that in controls, with some mice showing complete remission (Fig. 1J and K and Fig. S5A). Mice administered iAB-4T1 survived longer than the control groups (Fig. 1L), and the tumor weight of mice administered iAB-4T1 was significantly lower than that of control mice (Fig. S5B).

Other metastatic cancer models, CT-26-iRFP and B16-iRFP, were also constructed to evaluate the anticancer effect of iABs. The in vivo cancer models were created by administering CT-26-iRFP cells to the rectum and left flank, and B16-iRFP melanoma cells to both flanks. When the left flank of the CT-26-iRFP tumor and the right flank of the B16-iRFP tumors reached approximately 100 mm^3^ in size, the tumors were excised, and iABs were generated. Specifically, ABs were produced by treating cancer cells obtained from CT-26-iRFP and B16-iRFP tumors with Cis or Dox (Tables S3 to S6 and Figs. S6 and S7), and MPLA was inserted into ABs to form iABs. Notably, mice treated with CT-26-iRFP tumor-generated iABs (iAB-CT26) showed suppression of CT-26-iRFP tumor growth in the rectal area compared to that of tumors in control mice (Fig. 1M to O and Fig. S8). Similarly, iABs from B16-iRFP tumor cells (iAB-B16) effectively inhibited the growth of metastatic B16-iRFP melanoma (Fig. S9).

To confirm the treatment mechanism of residual metastatic cancer by iAB-CT26, mesenteric lymph nodes (mLNs) were harvested from CT-26-iRFP tumor-bearing mice. CD4 and CD8 T cells in the mLNs of mice treated with iAB-CT26 secreted tumor necrosis factor-α and interferon-γ with high efficiency in response to the tumor antigens (Fig. 1P and Fig. S10). Production of cytotoxic mediators in iAB-CT26-treated mLN CD8 T cells was also increased by tumor antigens (Fig. 1Q and Fig. S11). Furthermore, CD44^+^CD62L^−^ memory CD4 and CD8 T cells exhibited an increasing trend, whereas Foxp3^+^ regulatory CD4 T cells tended to decrease after iAB-CT26 treatment (Fig. 1R and S and Figs. S12 and S13). To assess cytotoxic T lymphocyte activation, CD8 T cells were isolated from the mLNs of iAB-CT26-treated mice and co-cultured with CT-26-iRFP cells (Fig. 1T), revealing that iAB-CT26-treated CD8 T cells effectively attacked CT-26-iRFP cells within a short time, whereas other control CD8 T cells did not (Fig. 1U and Videos S1 to S4). Additionally, administration of iAB-CT26 did not induce hepatotoxicity or inflammation of peripheral tissues (Figs. S14 and S15). Together, these data demonstrate that iAB treatment could promote antigen-specific T cell immunity.

To advance clinical trials, effective methods for selectively removing metastatic cancer from patients and for the large-scale production and storage of iABs will be essential. Particularly, in cases of deep-seated tumors or micrometastatic lesions, it is necessary to consider tumor acquisition strategies and methods for sufficient production of iABs from those tumors. Despite these limitations, iABs could offer several advantages. By leveraging patient-derived tumor material, it may be less affected by tumor heterogeneity and adaptable for tailored metastatic cancer treatment. Moreover, iABs could reduce the cost and time required to identify neo-antigens for developing cancer vaccines. Furthermore, by generating antigen-specific immune responses, iABs might elicit fewer acute inflammatory reactions than nonspecific immune checkpoint blockade. Collectively, these features suggest that iABs could represent a promising personalized therapeutic approach for metastatic cancer.

## Ethical Approval

All animal studies were performed with approval from the Institutional Animal Care and Use Committee of the ASAN Medical Center (approval no. 2023-04-041), and all experiments were performed after approval by a local ethics committee at the Laboratory Animals Center of ASAN Medical Center.

## Data Availability

The data that support the findings of this study are available from the corresponding author upon reasonable request.

## Supplementary Materials

Supplementary 1 Materials and Methods Tables S1 to S7 Figs. S1 to S15 Videos S1 to S4
