# Supplementary material for: Treatment of Metastatic Cancer by Conferring Immunogenicity to the Apoptotic Bodies of the Primary Tumor
Source: Cancer Commun (Lond). 2026 Jan 23;46:0001. doi: 10.34133/cancomm.0001 (PMC12856765; doi:10.34133/cancomm.0001)
Supplement: Supplementary 1 — Materials and Methods Tables S1 to S7 Figs. S1 to S15 Videos S1 to S4 [file cancomm.0001.f1.zip › CANCOMM-D-25-00117-Supplementary Material-Final.docx]

**Supplementary Materials**

**Materials and Methods**

**Cell lines**

The mouse breast cancer cell line 4T1-iRFP-Neo (4T1-iRFP) was purchased from Imanis Life Sciences (Rochester, MN, USA) and cultured in Roswell Park Memorial Institute-1640 medium (Sigma-Aldrich, St. Louis, MO, USA) containing 1% penicillin-streptomycin, 2 µg/mL puromycin (Sigma-Aldrich), and 10% fetal bovine serum (Sigma-Aldrich) in a 5% CO_2_ atmosphere at 37 °C. The mouse colon carcinoma cell line CT-26-iRFP-Neo (CT26-iRFP) was purchased from Imanis Life Sciences and cultured in Dulbecco's Modified Eagle Medium (Sigma-Aldrich) supplemented with 0.4 mg/mL G418 (Thermo Fisher Scientific, Waltham, MA, USA), 10% fetal bovine serum (Sigma-Aldrich), and 1% penicillin/streptomycin (Thermo Fisher Scientific) in a 5% CO_2_ cell incubator at 37 °C. The murine melanoma cell line B16F10-iRFP-Puro (B16-iRFP) was purchased from Imanis Life Sciences and cultured in Dulbecco's modified Eagle medium (Sigma-Aldrich) containing 1% penicillin-streptomycin, 2 µg/mL puromycin (Sigma-Aldrich), and 10% fetal bovine serum (Sigma-Aldrich) in a 5% CO_2_ atmosphere at 37°C.

**Mice**

Six- to eight-week-old BALB/c, C57BL/6, and TLR4-KO mice were obtained from Orient Bio, Inc. (Seongnam, Korea) and The Jackson Laboratory (Bar Harbor, ME, USA). Mice were housed under pathogen-free conditions at the Laboratory Animals Center of ASAN Medical Center. The protocol for the animal experiment was approved by the Institutional Animal Care and Use Committee of the ASAN Medical Center (approval no. 2023-04-041).

**Preparation of single suspended tumor cells from extracted tumors**

BALB/c mice were injected with 5 × 10^5^ 4T1-iRFP cells into the left and right fat pads. Eight days after injection, when the tumor size reached 91.31 ± 2.87 mm^3^, the right side of the tumor was surgically resected. In another model, 3 × 10^5^ CT-26-iRFP cells were administered into the rectum and left flank. On day 5 after injection, when the tumor size reached 97.69 ± 5.63 mm^3^, the left side of the tumor was surgically resected. In the other model, C57BL/6 mice were injected with 1 × 10^6^ B16-iRFP cells into the left and right flanks. On day 5 after injection, when the tumor size reached 103.21 ± 4.6 mm^3^, the right side of the tumor was surgically resected. Resected tumors were digested with a digestion buffer containing DNase and collagenase IV at 37°C for 10 min. Aggregated and undigested tissues were removed by filtration through a 100-nm nylon mesh. Finally, single suspended tumor cells were harvested after centrifugation at 590 ×g for 5 min.

**Induction of cell death and preparation of apoptotic bodies (ABs)**

4T1-iRFP and B16-iRFP cells obtained from surgically extracted tumors (1 × 10^6^ cells) were cultured in 6-well plates (SPL Life Sciences, Gyeonggi-do, Korea). The cells were treated with 15 or 30 μmol/L cisplatin (Sigma-Aldrich), 15 or 30 μmol/L doxorubicin (Sigma-Aldrich), or a combination of 15 μmol/L cisplatin and 15 μmol/L doxorubicin. At 24 h after treatment, ABs from 4T1-iRFP and B16-iRFP tumor cells were harvested. CT-26-iRFP cells obtained from surgically extracted tumors (1 × 10^6^ cells) were cultured in 6-well plates. The tumor cells were treated with 15 or 30 μmol/L cisplatin (Sigma-Aldrich), 15 or 30 μmol/L doxorubicin (Sigma-Aldrich), or a combination of 15 μmol/L cisplatin and 15 μmol/L doxorubicin. ABs from CT-26-iRFP tumor cells were harvested 48 h after treatment.

**Synthesis of immunogenic apoptotic bodies (iABs)**

ABs were filtered through a 10 μm filter to remove large apoptotic cells. The ABs were then centrifuged at 6,000 ×*g* for 10 min at 4°C to remove fragments of 0.5-2 μm. Next, 5 μm ABs were fixed with fixation buffer for 20 min, and then ABs and various concentrations of MPLA (Avanti Polar Lipids, Alabaster, AL, USA) were stirred at 25°C for 30 min. iABs were centrifuged at 6,000 ×*g* for 10 min at 4°C, and the supernatant was removed and re-dispersed in phosphate-buffered saline (PBS), followed by storage at 4°C until analysis. To confirm the MPLA insertion efficiency, the splenic DC activation ability of 40 μg MPLA and iABs inserted with 40 μg MPLA was compared, and iABs showed an activation efficiency of 97.2 ± 1.2 compared to MPLA. In addition, iABs did not show any change in the efficacy of splenic DC activation when it was stored at 4°C for a month.

**Measurement of iAB size and particle distribution**

Isolated ABs and iABs suspensions were analyzed using a Luna Automated Cell Counter (LUNA-ii™, Logos Biosystems, Annandale, VA, USA). The analysis settings were optimized and kept constant between samples.

**Morphological detection of iABs**

To detect the morphological changes of AB-4T1 after insertion of MPLA, AB-4T1 and iAB-4T1 were examined using Giemsa staining. The AB-4T1 and iAB-4T1 were attached to a slide glass using cytospin, fixed with methanol for 2 min, and dried. The samples were stained with Giemsa solution (Sigma-Aldrich) for 1 min and then washed with tap water. The samples on the slides were covered with a coverslip (Paul Marienfeld GmbH & Co.KG., Lauda-Königshofen, Germany) and imaged using a fluorescence microscope (EVOS M5000; Thermo Fisher Scientific).

**Coomassie Brilliant Blue Staining**

Protein samples, including 4T1-iRFP, CT-26-iRFP and B16-iRFP cell lysates, purified ABs, and iABs stored for varying durations (0, 1, 3, 5, and 10 days), were quantified using a BCA protein assay to ensure equal loading. Each sample (5–10 µg) was mixed with 4 × Laemmli sample buffer (Sigma-Aldrich) containing 5% β-mercaptoethanol (Sigma-Aldrich) and boiled at 95 °C for 5 min. Proteins were separated on a 10% SDS–polyacrylamide gel at 120 V until adequate resolution was achieved. Following electrophoresis, gels were stained with Coomassie brilliant blue R-250 solution (0.1% Coomassie brilliant blue R-250, 40% methanol, 10% acetic acid) for 1 h at 25 °C with gentle agitation. Gels were then destained using a solution of 40% methanol and 10% acetic acid, with several changes of destaining buffer until background staining was minimized. After a final rinse in deionized water, the gels were imaged. Band intensities were compared to assess the stability of iABs over the indicated storage periods.

**AB-4T1 and iAB-4T1 Labeling with Carboxyfluorescein Succinimidyl Ester (CFSE)**

AB-4T1 and iAB-4T1 were fluorescently labeled using the CellTrace™ CFSE Cell Proliferation Kit (Thermo Fisher Scientific). The working solution of the reagent was prepared according to the supplier's instructions and applied to the AB-4T1 and iAB-4T1 suspension under serum-free conditions to ensure uniform staining. After a brief incubation, the remaining reagent was neutralized in complete medium and gently washed to remove it. The labeled AB-4T1 and iAB-4T1 were cultured under standard culture conditions until use. Finally, the AB-4T1 and iAB-4T1 labeled with the stable green fluorescent CFSE signal was analyzed by fluorescence microscope (EVOS M5000) and NovoExpress (ACEA Biosciences, San Diego, CA, USA).

**iAB-4T1 uptake by bone marrow-derived dendritic cells (BMDCs)**

BMDCs were seeded at 2 × 10^5^ per well into 24-well plates. For BMDC differentiation, 100 ng/mL recombinant murine GM-CSF (R&D Systems, Minneapolis, MN, USA) and 100 ng/mL recombinant murine IL-4 (R&D Systems) were added to the medium. Cells were incubated for 6 days at 37°C in 10% CO_2_ in air, washed with fresh media, and then treated with CFSE-stained AB-4T1 and iAB-4T1. After 3 h of incubation, the medium was removed, and the samples were washed twice with PBS. CFSE-labled-AB-4T1 and CFSE-labeled-iAB uptake into BMDCs was analyzed using a fluorescence microscope (EVOS M5000) and NovoExpress (ACEA Biosciences) or FACS LSR II (BD Biosciences, Franklin Lakes, NJ, USA).

**Analysis of dendritic cell (DC) activation**

BALB/c mice were intravenously injected with PBS, AB-4T1, MPLA, and iAB-4T1. Spleens were harvested 18 h later. Briefly, the spleens were digested with a digestion buffer containing DNase and collagenase IV at 37°C for 10 min. Aggregated and undigested tissues were removed by filtration through a 100-nm nylon mesh. After washing with PBS, the pellet and upper layer were individually resuspended in 4 mL of Histopaque-1077 (Sigma-Aldrich). Next, medium containing fetal bovine serum was added to the upper layer of the cell suspension. The fraction exceeding 1.077 g/cm^3^ was centrifuged at 600 ×*g* for 10 min, and the leukocytes were collected and stained with CD11c lineage antibodies. CD11c^+^ lineage cells were subdivided into cDC1 and cDC2 based on CD8 expression. All antibodies used in this study are listed in Supplementary table S1.

**4T1-iRFP breast cancer model development and treatment**

BALB/c mice were injected with 5 × 10^5^ 4T1-iRFP cells into the left and right fat pads. On day 8 post-tumor induction, when the tumor reached a size of 91.31 ± 2.87mm^3^, the right side of the tumor was surgically resected. The surgical site was sutured with an automatic clip applier (Reflex7; Harvard Apparatus, Holliston, MA, USA). Two to five days after surgery, when the wound had healed, the clips were removed using a clip remover (Harvard Apparatus). The day after surgically removing the tumor, the mice were intraperitoneally administered PBS, AB-4T1, MPLA, and iAB-4T1 five times at 3-day intervals. Tumor volume was measured every 4 days and calculated using the formula V = 1/2 × (L×S^2^), where L is the longest dimension and S is the shortest dimension. 4T1-iRFP fluorescence expression levels were measured on days 7, 12, 18, and 22 after 4T1-iRFP tumor cell administration using an IVIS imaging system (Xenogen, Alameda, CA, USA).

**CT-26-iRFP colon cancer model development and treatment**

The BALB/c mice were injected with 3 × 10^5^ CT-26-iRFP cells into the rectum and left flank. Five days after tumor administration, the left flank tumor was excised when the size reached 97.69 ± 5.63 mm^3^, and the skin was sutured using an automatic clip applier (Harvard Apparatus) according to the same protocol as used in the 4T1-iRFP breast cancer model. The day after surgical tumor removal, the mice were intraperitoneally administered PBS, AB-CT26, MPLA, and iAB-CT26 five times at 2-day intervals. CT-26-iRFP fluorescence expression levels were measured on days 4, 7, 11, 15, and 18 after CT-26-iRFP tumor cell administration using an IVIS imaging system.

**B16-iRFP melanoma cancer model development and treatment**

C57BL/6 mice were injected with 1 × 10^6^ B16-iRFP cells into the left and right flanks. On day 5 post-tumor induction, when the tumor reached a size of 103.21 ± 4.6 mm^3^, the right side of the tumor was surgically resected and the skin was sutured using an automatic clip applier (Harvard Apparatus) following the same protocol of the 4T1-iRFP breast cancer model. The day after surgical tumor removal, the mice were intraperitoneally administered PBS, AB-B16, MPLA, and iAB-B16 five times at 2-day intervals. B16-iRFP fluorescence levels were measured on days 4, 7, 11, 15, and 18 after B16-iRFP tumor cell administration using an IVIS imaging system.

**Analysis of intracellular cytokine production in T cells**

Single cells from mLNs were harvested and stimulated with tumor cell lysate for 18 h. Monensin (BioLegend, San Diego, CA, USA) was added to cells 2 h before harvesting. After washing with PBS, the cells were stained with surface antibodies and fixed with 200 μL of fixation buffer (BioLegend) for 20 min. Finally, the cells were permeabilized with permeabilization buffer (BioLegend) and stained with intracellular cytokine antibodies for 20 min at 25℃. Surface and intracellular cytokine levels were analyzed using flow cytometry. All antibodies used in this study are listed in Supplementary table S1.

**CD8 T cell-mediated cytotoxicity**

For CD8 T cell isolation, iAB-CT26-stimulated mLNs were harvested, washed with PBS, and stained with anti-biotin microbeads (Miltenyi Biotec, Bergisch Gladbach, Germany), and negative cells were harvested as CD8 T cells. The isolated CD8 T cells (1 × 10^5^) were co-cultured with CT-26-iRFP cells (1 × 10^4^) in 48-well plates. CTL cytotoxicity against CT-26-iRFP cells was monitored for 15 h using a fluorescence microscope (EVOS M5000) and live cell incubator (LCIbio, Gyeonggi-do, Korea). All antibodies used in this study are listed in Supplementary table S7.

**Statistical analysis**

All data are expressed as the mean ± standard error of the mean (SEM). One- or two-way analysis of variance (ANOVA; Tukey multiple comparison test) was used to analyze the datasets. Significance of survival rates and tumor growth curve were determined by the log-rank test. Statistical significance was set at **P* < 0.05 and ***P* < 0.01 (SPSS software, version 23.0, SPSS, Chicago, IL, USA).

**Supplementary Table S1. Number of single suspended 4T1-iRFP tumor cells according to tumor size**

| 4T1-iRFP | **1** | **2** | **3** | **4** | **5** |
| --- | --- | --- | --- | --- | --- |
| **Tumor** | 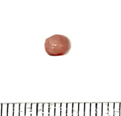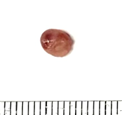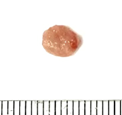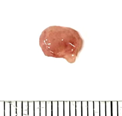 |  |  |  | 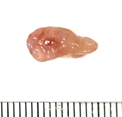 |
| **Volume (mm^3^)** | 34.38 ± 1.22 | 91.31 ± 2.87 | 171.05 ± 4.23 | 204.52 ± 1.54 | 308.79 ± 4.77 |
| **Weight (mg)** | 24.53 ± 3.50 | 45.88 ± 2.90 | 75.50 ± 3.92 | 107.00 ± 4.84 | 135.00 ± 2.89 |
| **Cell number (×10^6^)** | 3.01 ± 0.61 | 4.02 ± 0.38 | 5.62 ± 0.78 | 7.27 ± 0.83 | 8.11 ± 2.03 |

Different numbers of 4T1-iRFP cells were injected into BALB/c mice. Tumor tissues of different sizes were surgically extracted and digested with a digestion buffer. Single cells were analyzed using a Luna Automated Cell Counter (*n* = 6). The data is presented as mean ± standard error of the mean. Abbreviations: iRFP, near-infrared fluorescent protein.

**Supplementary Table S2. Number of apoptotic bodies obtained from 4T1-iRFP tumor cells treated with anti-cancer drugs**

| **Incubation for 24 h** | **1 × 10^6^ AB-4T1 number/1 × 10^6^ cells** |
| --- | --- |
| **Cis 15 μmol/L** | 0.21 ± 0.01 |
| **Cis 30 μmol/L** | 0.37 ± 0.11 |
| **Dox 15 μmol/L** | 0.64 ± 0.17 |
| **Dox 30 μmol/L** | 0.83 ± 0.23 |
| **Cis 15 μmol/L + Dox 15 μmol/L** | 1.35 ± 0.20 |

4T1-iRFP cells obtained from surgically extracted tumors (1 × 10^6^ cells) were treated with 15 or 30 μmol/L cisplatin (Cis), 15 or 30 μmol/L doxorubicin (Dox), or a combination of 15 μmol/L Cis and 15 μmol/L Dox for 24 h. AB-4T1 were analyzed using a Luna Automated Cell Counter (*n* = 6). The data is presented as mean ± standard error of the mean. Abbreviations: AB, apoptotic body; iRFP, near-infrared fluorescent protein; Cis, cisplatin; Dox, doxorubicin.

**Supplementary Table S3. Number of single suspended CT-26-iRFP tumor cells according to tumor size**

| **CT-26-iRFP** | **1** | **2** | **3** | **4** | **5** |
| --- | --- | --- | --- | --- | --- |
| **Tumor** | 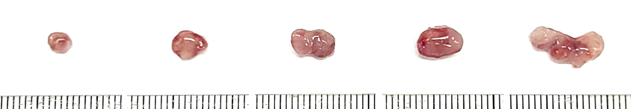 | 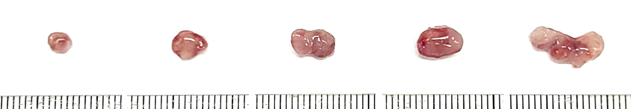 | 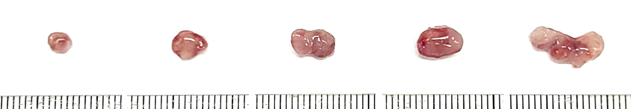 | 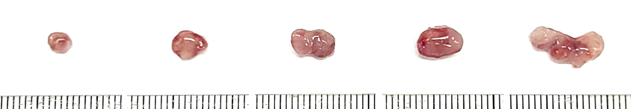 | 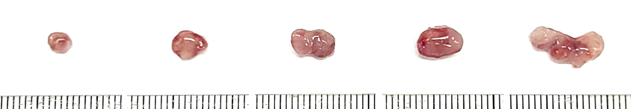 |
| **Volume (mm^3^)** | 35.50 ± 3.27 | 97.69 ± 5.63 | 135.00 ± 3.33 | 199.36 ± 8.66 | 308.20 ± 5.90 |
| **Weight (mg)** | 30.20 ± 2.88 | 78.27 ± 1.56 | 89.70 ± 3.01 | 113.00 ± 5.11 | 206.02 ± 3.80 |
| **Cell number (×10^6^)** | 2.65 ± 0.11 | 3.52 ± 0.89 | 7.02 ± 0.93 | 10.43 ± 1.30 | 12.60 ± 0.93 |

Different numbers of CT-26-iRFP cells were injected into BALB/c mice. Tumor tissues of different sizes were surgically extracted and digested with a digestion buffer. Single cells were analyzed using a Luna Automated Cell Counter (*n* = 6). The data is presented as mean ± standard error of the mean. Abbreviations: iRFP, near-infrared fluorescent protein.

**Supplementary Table S4.** **Number of apoptotic bodies obtained from CT-26-iRFP tumor cells treated with anticancer drugs**

| **Incubation for 24 h** | **1 × 10^5^ AB-CT26 number/1 × 10^6^ cells** | **AB-CT26 size (μm)** |
| --- | --- | --- |
| **Cis 15 μmol/L** | NA | NA |
| **Cis 30 μmol/L** | 0.05 ± 0.02 | 6.2 |
| **Dox 15 μmol/L** | 0.41 ± 0.03 | 6.9 |
| **Dox 30 μ mol/L** | 0.61 ± 0.06 | 5.1 |
| **Cis 15 μmol/L + Dox 15 μmol/L** | 0.70 ± 0.08 | 5.7 |
| **Incubation for 48 h** | **1 × 10^6^ AB-CT26 number/1 × 10^6^ cells** | **AB-CT26 size (μm)** |
| **Cis 15 μmol/L** | 0.12 ± 0.02 | 5.3 |
| **Cis 30 μmol/L** | 0.17 ± 0.01 | 5.1 |
| **Dox 15 μmol/L** | 2.76 ± 0.13 | 5.3 |
| **Dox 30 μmol/L** | 3.87 ± 0.06 | 5.3 |
| **Cis 15 μmol/L + Dox 15 μmol/L** | 2.26 ± 0.04 | 5.5 |

CT-26-iRFP cells obtained from a surgically extracted tumor (1 × 10^6^ cells) were treated with 15 or 30 μmol/L cisplatin (Cis), 15 or 30 μmol/L doxorubicin (Dox), or a combination of 15 μmol/L Cis and 15 μmol/L Dox for 24 and 48 h. AB-CT26 were analyzed using a Luna Automated Cell Counter (*n* = 6). The data is presented as mean ± standard error of the mean. Abbreviations: AB, apoptotic body; Cis, cisplatin; Dox, doxorubicin; iRFP, near-infrared fluorescent protein; NA, Not Available.

**Supplementary Table S5. Number of single suspended B16-iRFP tumor cells according to tumor size**

| **B16-iRFP** | **1** | **2** | **3** | **4** | **5** |
| --- | --- | --- | --- | --- | --- |
| **Tumor** | 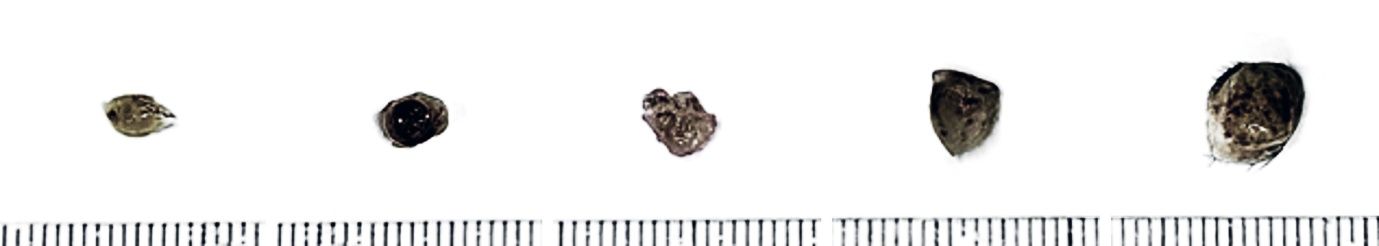 | 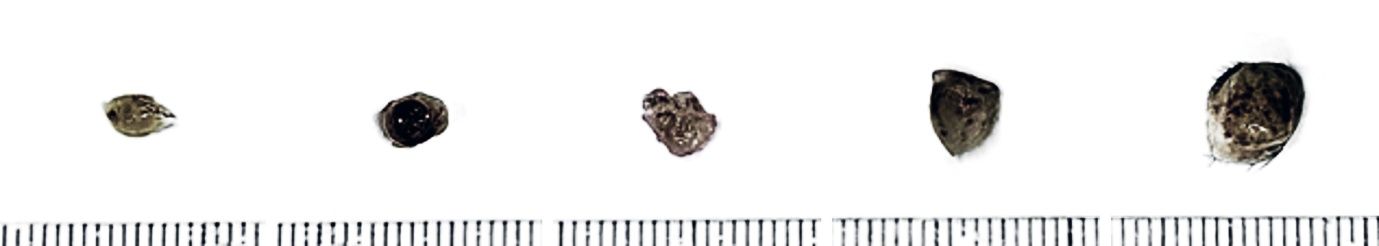 | 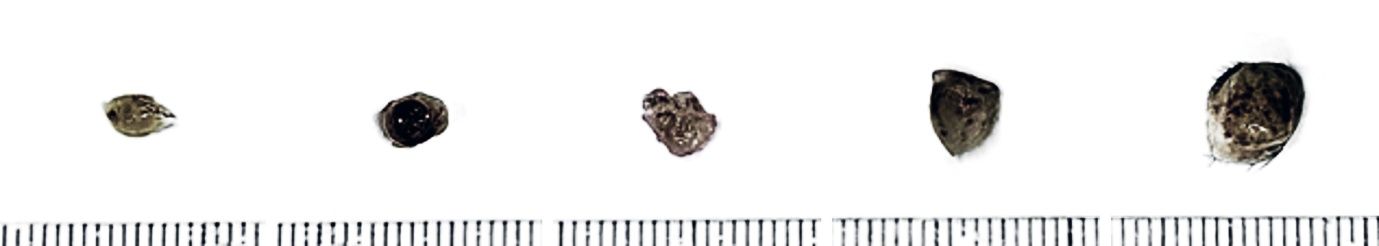 | 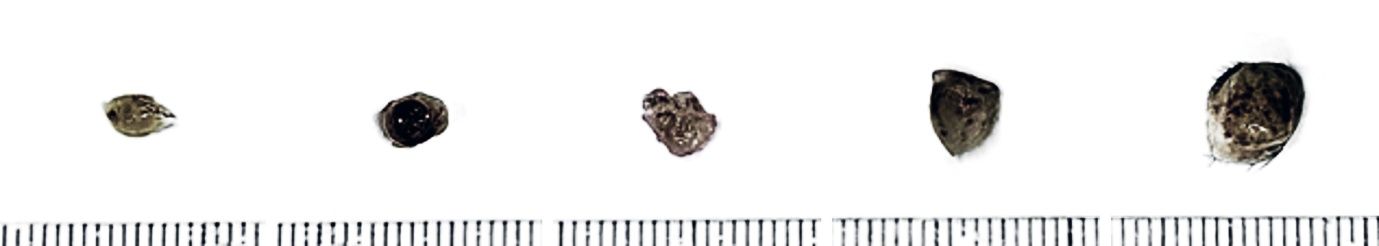 | 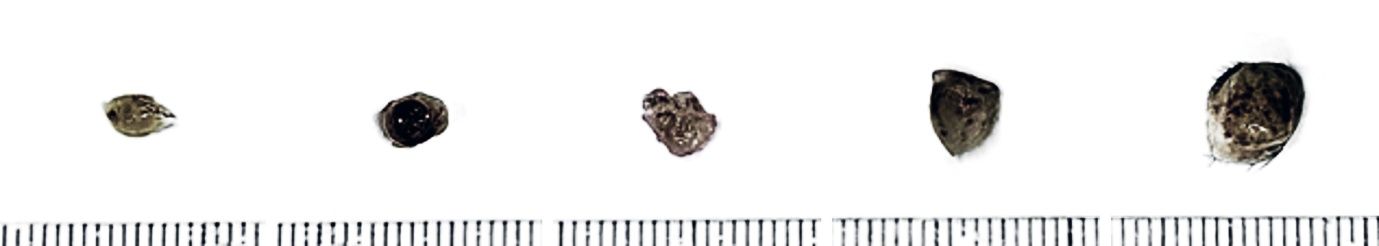 |
| **Volume (mm^3^)** | 33.44 ± 6.08 | 103.21 ± 4.60 | 124.33 ± 7.80 | 163.11 ± 6.77 | 298.11 ± 8.59 |
| **Weight (mg)** | 23.40 ± 4.30 | 71.50 ± 2.10 | 90.10 ± 3.50 | 128.09 ± 5.52 | 354.00 ± 5.12 |
| **Cell number (×10^6^)** | 2.55 ± 0.13 | 4.01 ± 0.32 | 5.87 ± 0.97 | 6.72 ± 1.32 | 8.41 ± 1.25 |

Different numbers of B16-iRFP cells were injected into C57BL/6 mice. Tumor tissues of different sizes were surgically extracted and digested with a digestion buffer. Single cells were analyzed using a Luna Automated Cell Counter (*n* = 6). The data is presented as mean ± standard error of the mean. Abbreviations: iRFP, near-infrared fluorescent protein.

**Supplementary Table S6. Number of apoptotic bodies obtained from B16-iRFP tumor cells treated with anticancer drugs**

| **Incubation for 24 h** | **1 × 10^6^ AB-B16 number/1 × 10^6^ cells** |
| --- | --- |
| **Cis 15 μmol/L** | 0.10 ± 0.06 |
| **Cis 30 μmol/L** | 0.18 ± 0.02 |
| **Dox 15 μmol/L** | 2.32 ± 0.36 |
| **Dox 30 μmol/L** | 5.28 ± 0.57 |
| **Cis 15 μmol/L + Dox 15 μmol/L** | 2.22 ± 0.30 |

B16-iRFP cells obtained from surgically extracted tumors (1 × 10^6^ cells) were treated with 15 or 30 μmol/L cisplatin (Cis), 15 or 30 μmol/L doxorubicin (Dox), or a combination of 15 μmol/L Cis and 15 μmol/L Dox for 24 h. AB-B16 were analyzed using a Luna Automated Cell Counter (*n* = 6). The data is presented as mean ± standard error of the mean. Abbreviations: AB, apoptotic body; Cis, cisplatin; Dox, doxorubicin; iRFP, near-infrared fluorescent protein.

**Supplementary Table S7. Antibodies used in the study**

| **Antibody** | **Conjugation** | **Clone number** | **Manufacturer** | **Application** |
| --- | --- | --- | --- | --- |
| Anti-mouse CD90.1 | FITC | S20007C | BioLegend | Flow cytometry |
| Anti-mouse CD3 | FITC | 17A2 | BioLegend | Flow cytometry |
| TER-119/erythroid | FITC | TER-119 | BioLegend | Flow cytometry |
| Anti-mouse CD45R/B220 | FITC | RA3-6B2 | BioLegend | Flow cytometry |
| Anti-mouse CD40 | APC | 3/23 | BioLegend | Flow cytometry |
| Anti-mouse Ly-6G/Ly-6C | FITC | RB6-8C5 | BioLegend | Flow cytometry |
| Anti-mouse CD80 | Brilliant Violet 605™ | 16-10A1 | BioLegend | Flow cytometry |
| Anti-mouse CD49b | FITC | DX5 | BioLegend | Flow cytometry |
| Anti-mouse CD11c | Brilliant Violet 785™ | N418 | BioLegend | Flow cytometry |
| Anti-mouse H-2Kb | PerCP/cyanine5.5 | AF6-88.5 | BioLegend | Flow cytometry |
| Anti-mouse CD86 | PE/cyanine7 | GL-1 | BioLegend | Flow cytometry |
| Anti-mouse I-A/I-E | PerCP/cyanine5.5 | M5/114.15.2 | BioLegend | Flow cytometry |
| Anti-mouse CD8a | APC/cyanine7 | 53-6.7 | BioLegend | Flow cytometry |
| Anti-mouse CD4 | PE/cyanine5 | RM4-5 | BioLegend | Flow cytometry |
| Anti-mouse TCR-β | PE/cyanine7 | H57-597 | BioLegend | Flow cytometry |
| Anti-mouse IFN-γ | PE/cyanine7 | XMG1.2 | BioLegend | Flow cytometry |
| Anti-mouse perforin | PE | S16009A | BioLegend | Flow cytometry |
| Anti-mouse granzyme B | APC | QA16A02 | BioLegend | Flow cytometry |
| Anti-mouse TNF-α | FITC | MP6-XT22 | BioLegend | Flow cytometry |
| Anti-mouse CD11b | Biotin | M1/70 | BioLegend | CD8 T cell isolation |
| Anti-mouse CD11c | Biotin | N418 | BioLegend | CD8 T cell isolation |
| Anti-mouse CD45R/B220 | Biotin | RA3-6B2 | BioLegend | CD8 T cell isolation |
| Anti-mouse CD19 | Biotin | MB19-1 | BioLegend | CD8 T cell isolation |
| Anti-mouse CD49b | Biotin | DX5 | BioLegend | CD8 T cell isolation |
| Anti-mouse TER-119/erythroid | Biotin | TER-119 | BioLegend | CD8 T cell isolation |
| Anti-mouse CD25 | Biotin | PC61 | BioLegend | CD8 T cell isolation |
| Anti-mouse CD4 | Biotin | RM4-5 | BioLegend | CD8 T cell isolation |
| Anti-mouse CD122 | Biotin | TM-β1 | BioLegend | CD8 T cell isolation |


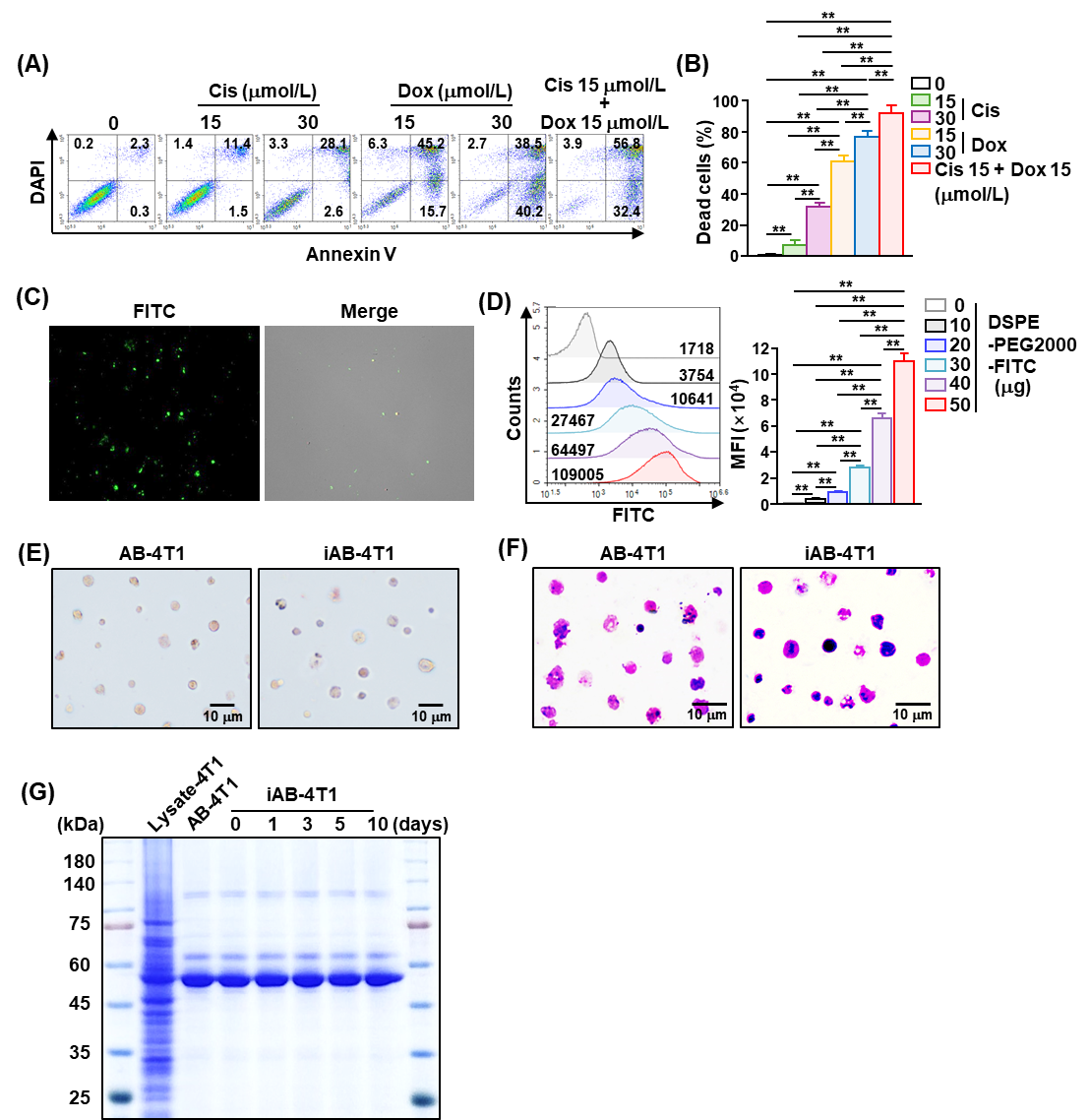


**Supplementary Figure S1. Production of immunogenic apoptotic bodies (ABs). (A)** The 4T1-iRFP cells were treated with 15 or 30 μmol/L cisplatin (Cis), 15 or 30 μmol/L doxorubicin (Dox), or a combination of 15 μmol/L Cis and 15 μmol/L Dox. Apoptotic and necrotic cell death of the cells was measured by Annexin V/DAPI staining. **(B)** Mean dead cells of 4T1-iRFP after treatment with Cis or Dox (*n*= 6, two-way ANOVA, mean ± SEM, ***P* < 0.01). **(C)** Insertion of DSPE-PEG2000-FITC (40 μg) into AB-4T1 was analyzed using a fluorescence microscopy. **(D)** FITC fluorescence intensity of AB-4T1 following insertion of DSPE-PEG2000-FITC. The numbers indicated mean fluorescence intensity (MFI) of FITC in the AB-4T1 (left panel). The graph showed the MFI of FITC fluorescence in AB-4T1 (right panel). **(E)** Microscope images of AB-4T1 and iAB-4T1 immediately after production. **(F)** Giemsa-stained image of AB-4T1 and iAB-4T1 immediately after production. **(G)** Coomassie staining for measuring protein changes in iAB-4T1 during incubation time. Abbreviations: AB, apoptotic body; ANOVA, analysis of variance; Cis, cisplatin; DAPI, 4′,6-diamidino-2-phenylindole; Dox, doxorubicin; DSPE-PEG2000-FITC, 1,2-distearoyl-*sn*-glycero-3-phosphoethanolamine-N-[poly(ethyleneglycol)-2000]-N-(fluorescein isothiocyanate); iAB, immunogenic apoptotic body; iRFP, near-infrared fluorescent protein; MFI, mean fluorescence intensity; SEM, standard error of the mean.


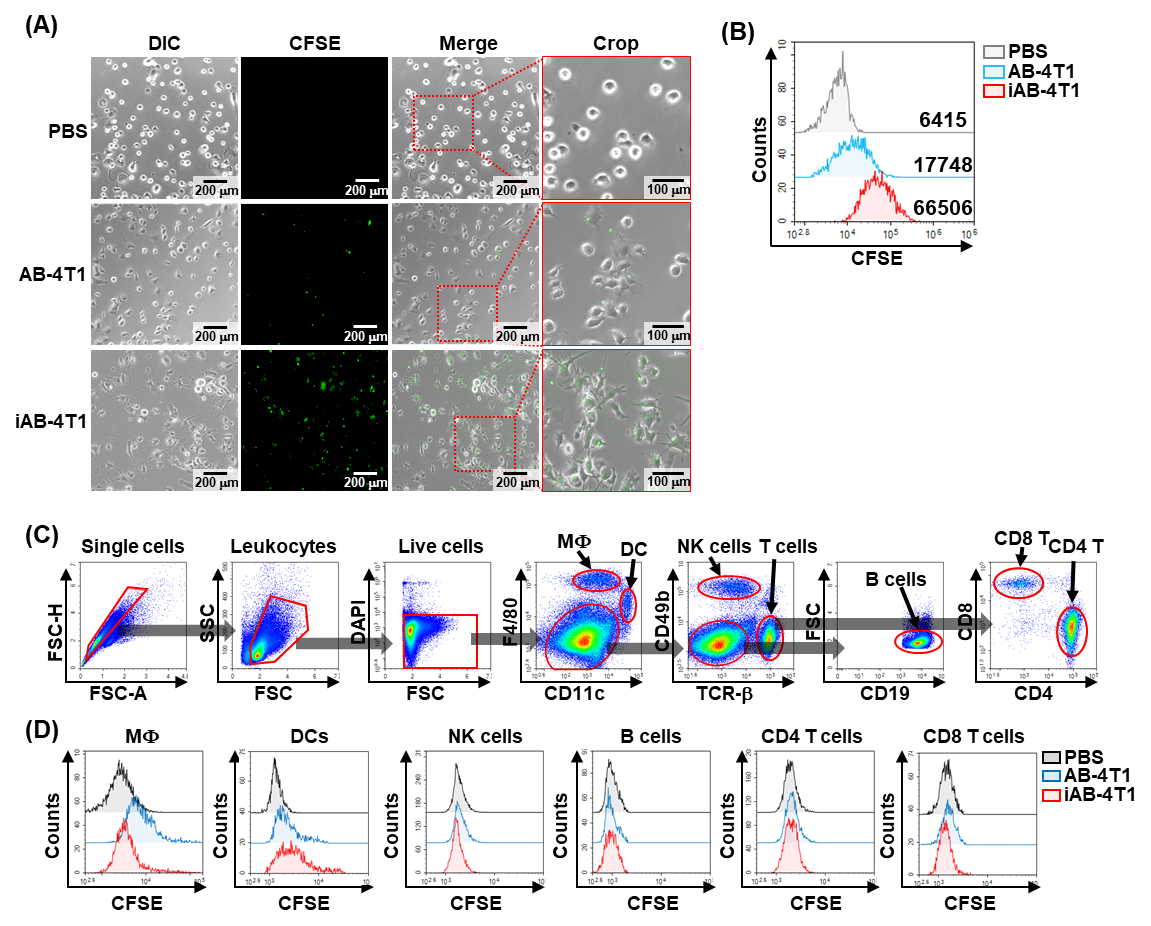


**Supplementary Figure S2. Targeting splenic DCs using iAB-4T1.** **(A)** Bone marrow-derived dendritic cells (BMDCs) were incubated with carboxyfluorescein succinimidyl ester (CFSE)-labeled AB-4T1 or iAB-4T1. Three hours after incubation, the cells were observed under a fluorescence microscope. **(B)** CFSE fluorescence was analyzed in BMDCs by treatment with PBS, CFSE-labeled AB-4T1 and CFSE-labeled iAB-4T1. The numbers indicated MFI of CFSE in the BMDCs. **(C)** Spleen immune cell compartmentalization using flow cytometry. **(D)** Analysis of CSFE fluorescence taken up from the indicated immune cells was detected using flow cytometry. Abbreviations: AB, apoptotic body; BMDC, bone marrow-derived dendritic cell; CD, cluster of differentiation; CFSE, Carboxyfluorescein succinimidyl ester; DC, dendritic cell; DIC, Differential Interference Contrast; iAB, immunogenic apoptotic body; MΦ, macrophage; NK, natural killer; PBS, phosphate-buffered saline.


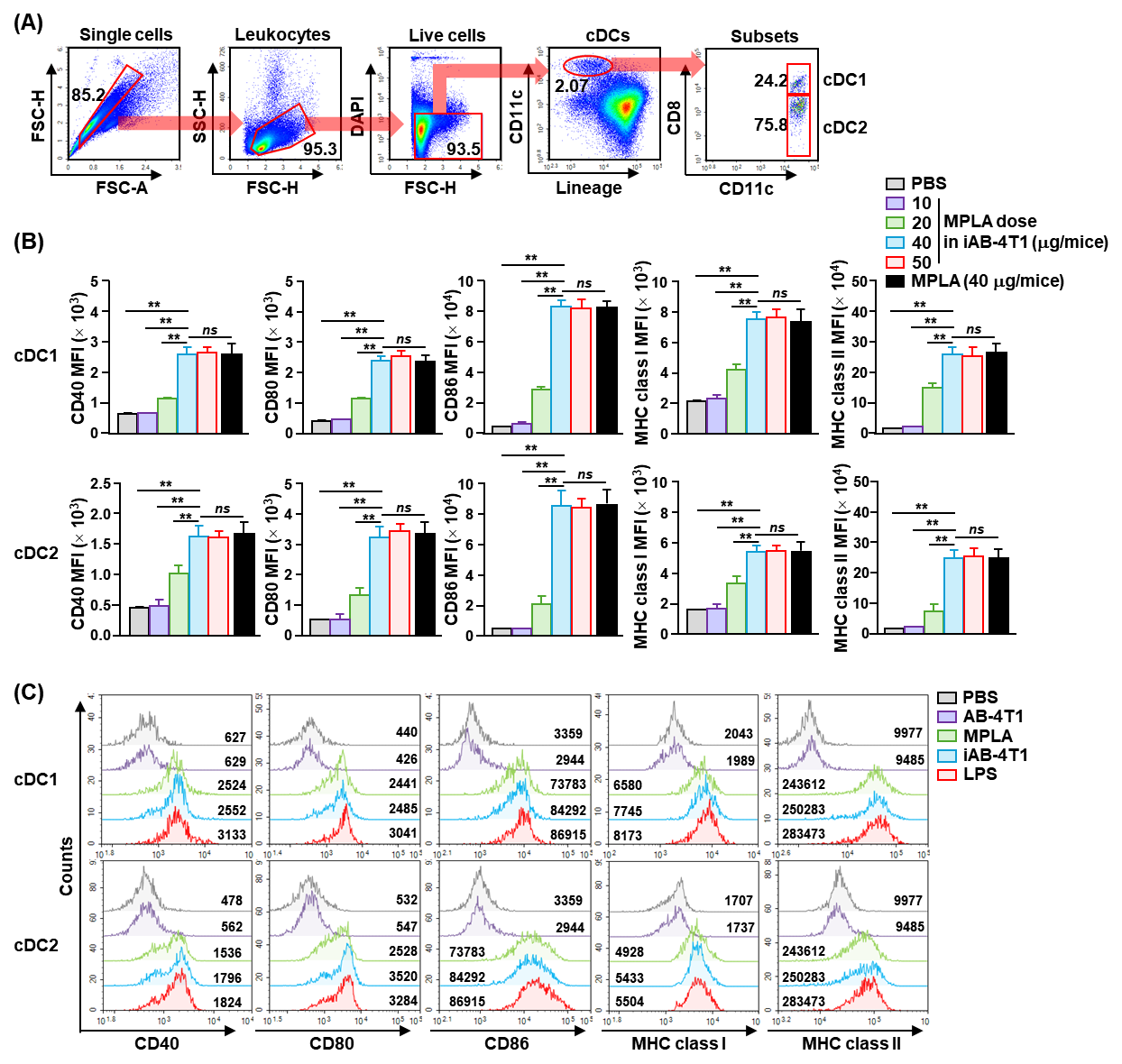


**Supplementary Figure S3. Activation of splenic DCs by iAB-4T1**. BALB/c mice were injected intravenously with PBS, AB-4T1, MPLA, iAB-4T1, and LPS. Eighteen hours after injection, the spleen was harvested, and cells were stained with a lineage marker and CD11c. **(A)** Gating strategy of splenic cDC1 and cDC2. **(B)** CD40, CD80, CD86, and MHC class I and II levels were analyzed in splenic cDC1 (upper panel) and cDC2 (lower panel) 18 h after injection of dose-dependent MPLA inserted iAB-4T1. MPLA (40 μg) was administered to mice as a positive control. (*n* = 6 mice, two-way ANOVA, mean ± SEM). **(C)** Surface activation markers and MHC molecule levels in cDC1 (upper panel) and cDC2 (lower panel) 18 h after injection of PBS, AB-4T1, MPLA, iAB-4T1, and LPS. The numbers represented MFI of indicated markers in the splenic DCs. Abbreviations: ANOVA, analysis of variance; AB, apoptotic body; CD, cluster of differentiation; cDC1, conventional type 1 DC; cDC2, conventional type 2 DC; DCs, dendritic cells; PBS, phosphate-buffered saline; iAB, immunogenic apoptotic body; LPS, lipopolysaccharide; MHC, major histocompatibility complex; MPLA, monophosphoryl lipid A; SEM, standard error of the mean.


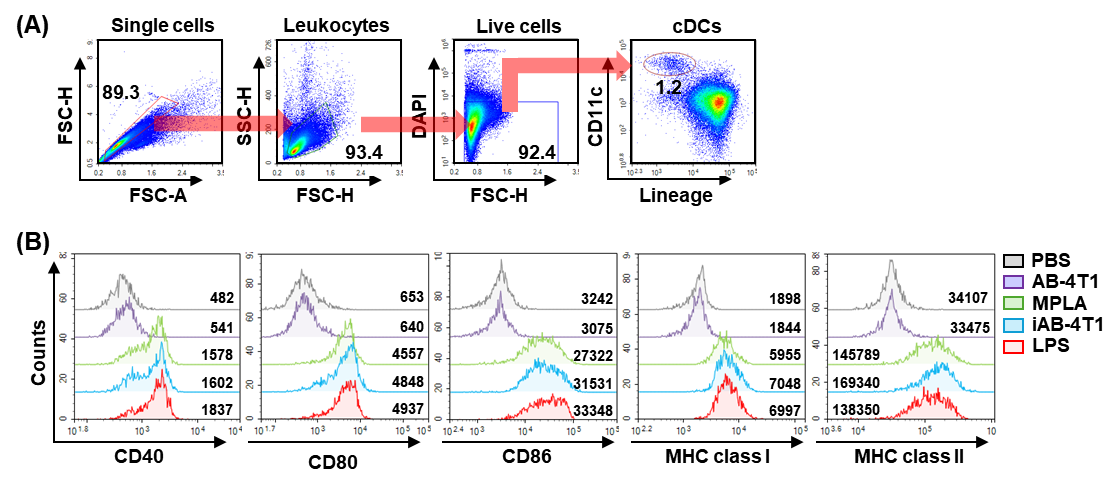


**Supplementary Figure S4. Activation of tumor draining lymph nodes (tdLNs) DCs by iAB-4T1.** BALB/c mice were injected intravenously with PBS, AB-4T1, MPLA, iAB-4T1, and LPS. Eighteen hours after injection, tdLNs were harvested. **(A)** Gating strategy of tdLN DCs. **(B)** Surface activation markers and MHC molecule levels in tdLN DCs 18 h after injection of PBS, AB-4T1, MPLA, iAB-4T1, and LPS. The numbers represented MFI of indicated markers in the tdLN DCs. Abbreviations: AB, apoptotic body; cDC1, conventional type 1 DC; cDC2, conventional type 2 DC; DCs, dendritic cells; iAB, immunogenic apoptotic body; LPS, lipopolysaccharide; MHC, major histocompatibility complex; MPLA, monophosphoryl lipid A; PBS, phosphate-buffered saline; SEM, standard error of the mean; tdLNs, tumor-draining lymph nodes.


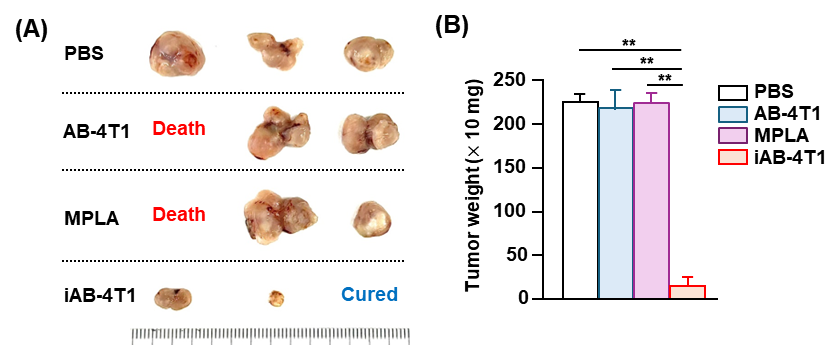


**Supplementary Figure S5. Inhibition of 4T1-iRFP tumor growth by iAB-4T1. (A)** Representative tumor mass from treated mice. **(B)** Mean tumor weight (*n* = 6, ***P* < 0.01, two-way ANOVA, mean ± SEM). Abbreviations: ANOVA, analysis of variance; AB, apoptotic body; iAB, immunogenic apoptotic body; MPLA, monophosphoryl lipid A; PBS, phosphate-buffered saline; SEM, standard error of the mean.


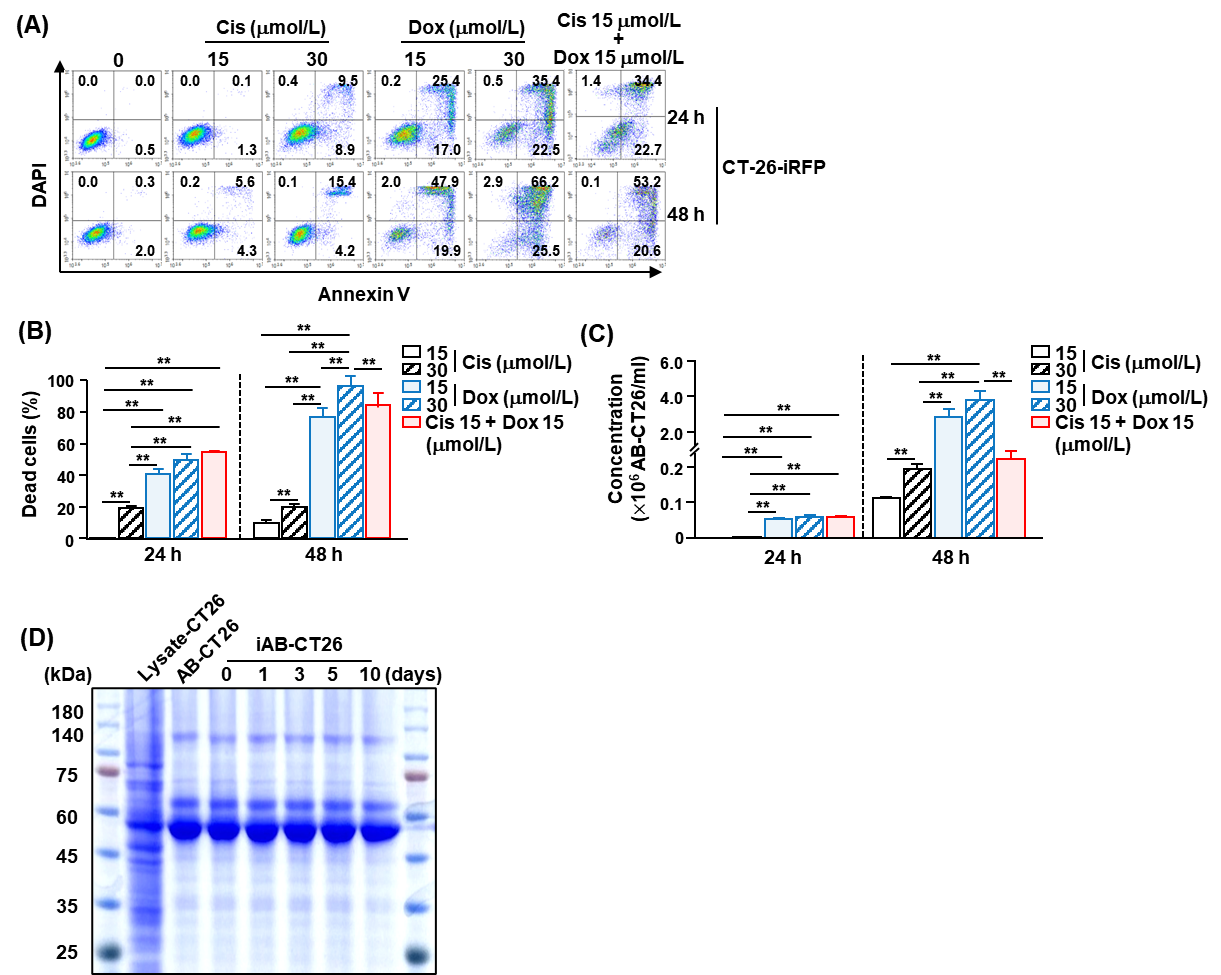


**Supplementary Figure S6. Number of ABs formed in CT-26-iRFP tumor cells treated with anticancer drugs.** CT-26-iRFP cells obtained from a surgically extracted tumor (1 × 10^6^ cells) were treated with 15 or 30 μmol/L cisplatin (Cis), 15 or 30 μmol/L doxorubicin (Dox), or a combination of 15 μmol/L Cis and 15 μmol/L Dox for 24 and 48 h. **(A)** Induction of apoptosis was analyzed by annexin V/DAPI staining. **(B)** Mean dead cells after treatment (*n* = 6, ***P* < 0.01, two-way ANOVA, mean ± SEM). **(C)** Number of AB-CT26 was analyzed using a Luna Automated Cell Counter (*n* = 6, ***P*< 0.01, two-way ANOVA, mean ± SEM). **(D)** Coomassie staining for measuring protein changes in AB-CT26 during incubation time. Abbreviations: ANOVA, analysis of variance; AB, apoptotic body; Cis, cisplatin; DAPI, 4′,6-diamidino-2-phenylindole; Dox, doxorubicin; iAB, immunogenic apoptotic body; iRFP, near-infrared fluorescent protein; SEM, standard error of the mean.


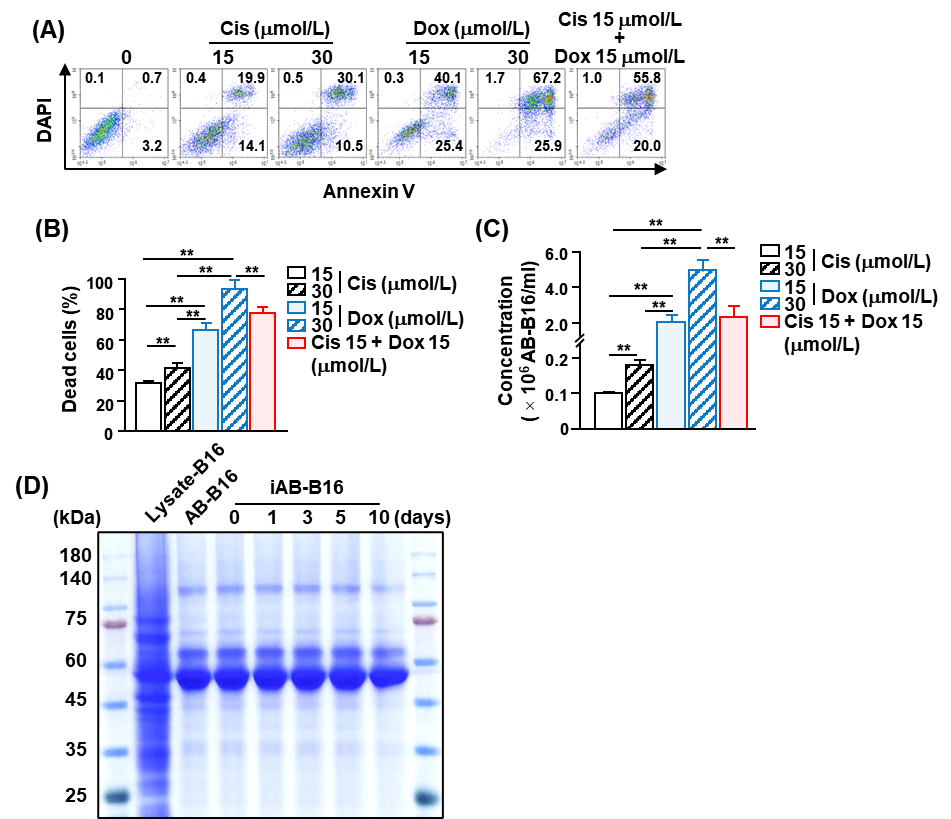


**Supplementary Figure S7. Number of AB-B16 formed in B16-iRFP tumor cells treated with anticancer drugs.** B16-iRFP cells were administered into the left and right flank. Five days after tumor administration, when the size of the right flank tumor reached 103.21 ± 4.6 mm^3^, the tumor was excised, and the single cells were treated with 15 or 30 μmol/L cisplatin (Cis), 15 or 30 μmol/L doxorubicin (Dox), or a combination of 15 μmol/L Cis and 15 μmol/L. **(A)** Induction of apoptosis was analyzed. **(B)** Mean dead cells (*n* = 6, ***P* < 0.01, two-way ANOVA, mean ± SEM). **(C)** Production number of iAB-B16 after treatment with cis or dox (*n* = 6, ***P* < 0.01, two-way ANOVA, mean ± SEM). **(D)** Coomassie staining for measuring protein changes in AB-B16 during incubation time. Abbreviations: ANOVA, analysis of variance; AB, apoptotic body; Cis, cisplatin; DAPI, 4′,6-diamidino-2-phenylindole; Dox, doxorubicin; iAB, immunogenic apoptotic body; iRFP, near-infrared fluorescent protein; SEM, standard error of the mean.


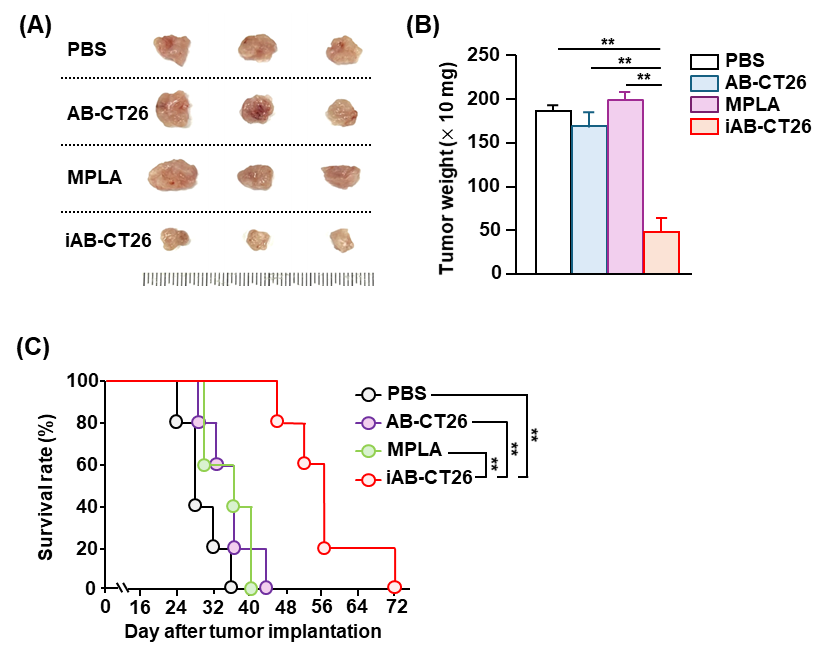


**Supplementary Figure S8. Suppression of CT-26-iRFP tumor growth by iAB-CT26.** CT-26-iRFP cells were administered into the rectum and left flank. Five days after tumor administration, when the size of the left flank tumor reached 97.69 ± 5.63 mm^3^, the tumor was excised, and iAB-CT26 were produced. PBS, AB-CT26, MPLA, and iAB-CT26 were administered at 2-day intervals starting 7 days after tumor administration, and tumor growth was observed. **(A)** Size of the extracted tumor mass. **(B)** Mean tumor weight (*n* = 6, ***P* < 0.01, two-way ANOVA, mean ± SEM). **(C)** Survival rate after drug treatment in CT-26-iRFP tumor-bearing mice (*n* = 5 mice, significance was determined by the log-rank test, mean ± SEM, ***P* < 0.01). Abbreviations: ANOVA, analysis of variance; AB, apoptotic body; iAB, immunogenic apoptotic body; iRFP, near-infrared fluorescent protein; MPLA, monophosphoryl lipid A; PBS, phosphate-buffered saline; SEM, standard error of the mean.


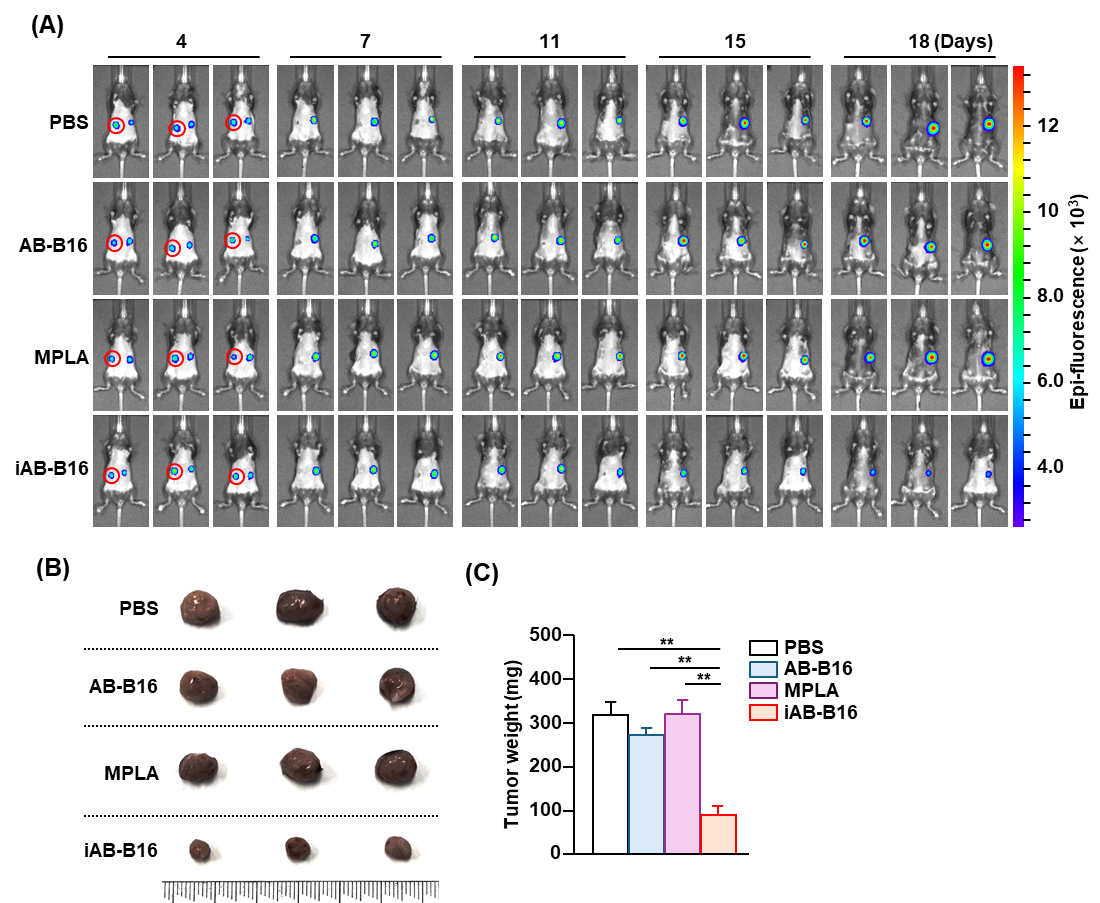


**Supplementary Figure S9. Suppression of B16-iRFP tumor growth by iAB-B16.** PBS, AB-B16, MPLA, and iAB-B16 were administered at 2-day intervals starting 2 days after surgical removal of the right tumor, and tumor growth was observed. **(A)** Fluorescence-based time-dependent tumor growth of B16-iRFP. **(B)** Size of the extracted tumor mass. **(C)** Mean tumor weight (*n* = 6, ***P* < 0.01, two-way ANOVA, mean ± SEM). Abbreviations: ANOVA, analysis of variance; AB, apoptotic body; iAB, immunogenic apoptotic body; iRFP, near-infrared fluorescent protein; MPLA, monophosphoryl lipid A; PBS, phosphate-buffered saline; SEM, standard error of the mean.


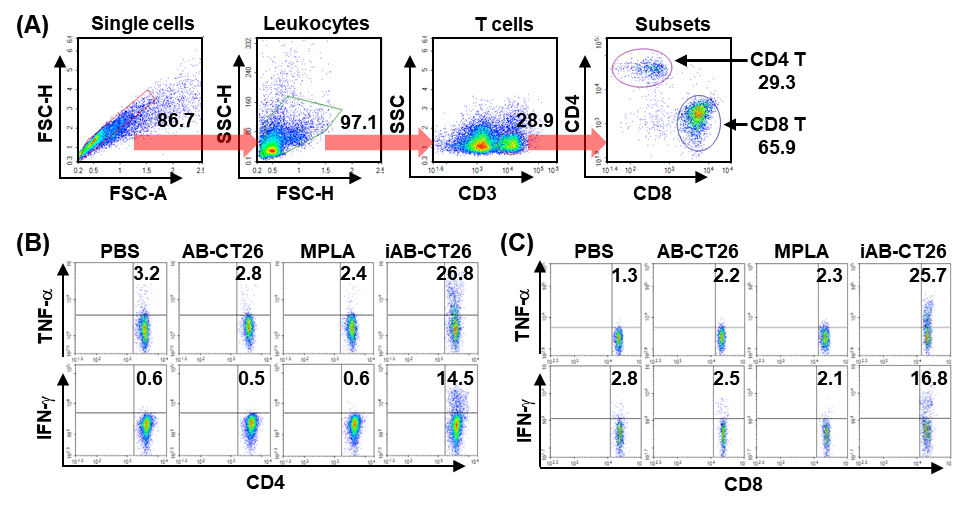


**Supplementary Figure S10. Definition of CD4 and CD8 T cells in** **mesenteric lymph nodes (mLNs).** The mLN CD4 T cells were defined as CD3^+^CD4^+^, and mLN CD8 T cells were defined as CD3^+^CD8^+^ cells in live leukocytes. **(A)** Gating strategy of mLN CD4 and CD8 T cells. **(B)** Intracellular levels of TNF-α and IFN-γ produced in mLN CD4 T cells. The numbers shown in the dot plot represent the positive percentage. **(C)** Intracellular levels of TNF-α and IFN-γ produced in mLN CD8 T cells. The numbers shown in the dot plot represented the positive percentages of cells. Abbreviations: AB, apoptotic body; CD, cluster of differentiation; IFN-γ, interferon-γ; iAB, immunogenic apoptotic body; mLN, mesenteric lymph node; MPLA, monophosphoryl lipid A; PBS, phosphate-buffered saline; TNF-α, tumor necrosis factor-α.


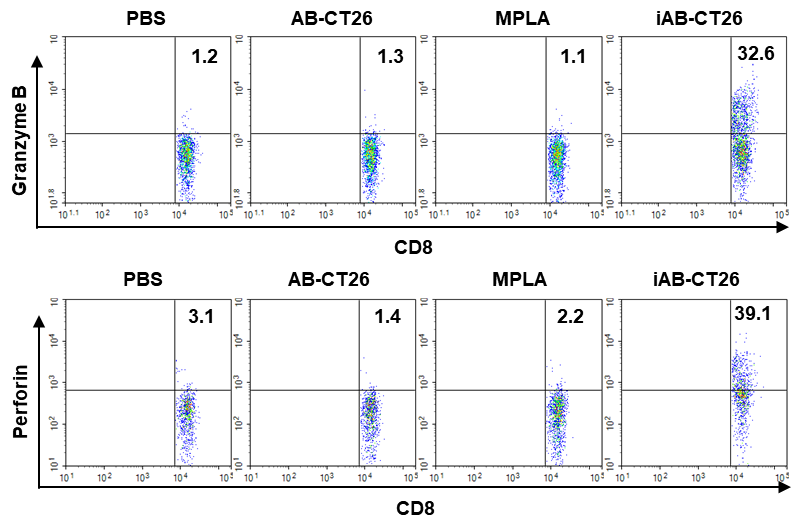


**Supplementary Figure S11. CT-26-iRFP antigen-specific cytokine production in mesenteric lymph node (mLN) cells.** Intracellular levels of granzyme B (upper panel) and perforin (lower panel) produced in mLN CD8 T cells. The numbers shown in the dot plot represented the positive percentages of cells. Abbreviations: AB, apoptotic body; CD: cluster of differentiation; iAB, immunogenic apoptotic body: iRFP, near-infrared fluorescent protein; mLN, mesenteric lymph node; MPLA, monophosphoryl lipid A; PBS, phosphate-buffered saline.


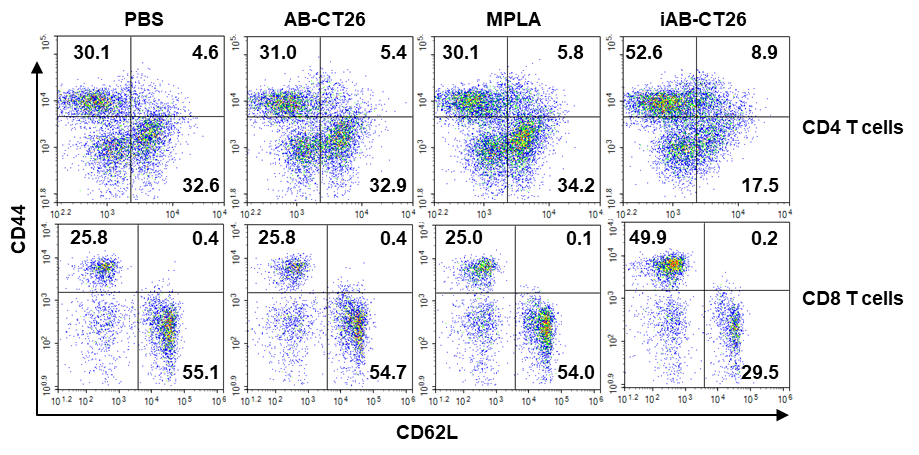


**Supplementary Figure S12. Definition of memory T cells in mesenteric lymph nodes (mLNs).** CD4 and CD8 T cells were distinguished from the CD3^+^ live cells in mLNs. CD44^+^CD62L^-^ memory cells in CD4 (upper panel) and CD8 T cells (lower panel). The numbers shown in the dot plot represented the positive percentages of cells. Abbreviations: AB, apoptotic body; CD, cluster of differentiation; iAB, immunogenic apoptotic body; mLN, mesenteric lymph node; MPLA, monophosphoryl lipid A; PBS, phosphate-buffered saline.


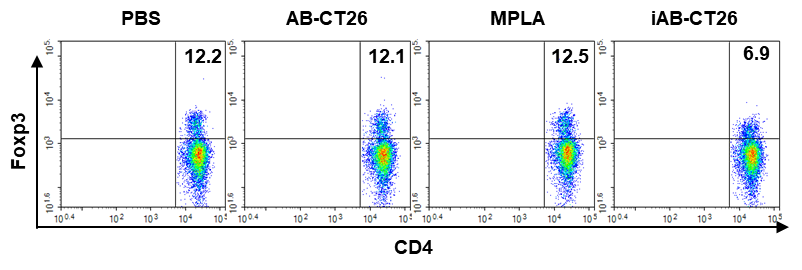


**Supplementary Figure S13. Definition of Foxp3^+^ regulatory T cells in mesenteric lymph nodes (mLNs).** Foxp3^+^ regulatory T cells were distinguished from the CD3^+^ and CD4^+^ live cells in mLNs. Foxp3^+^ cells in CD4 T cells was analyzed. The numbers shown in the dot plot represented the positive percentages of cells. Abbreviations: AB, apoptotic body; CD, cluster of differentiation; iAB, immunogenic apoptotic body; MPLA, monophosphoryl lipid A; mLN, mesenteric lymph node; PBS, phosphate-buffered saline.


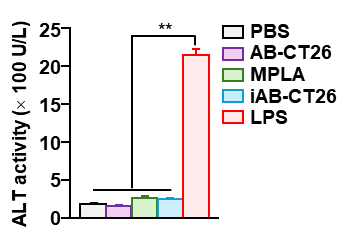


**Supplementary Figure S14.** **Liver toxicity of iAB-CT26.** BALB/c mice were intraperitoneally administered PBS, AB-CT26, MPLA, iAB-CT26, and LPS at 2-day intervals starting 7 days after tumor administration. LPS was used as a positive control for inducing hepatotoxicity. At 24 h after the last injection, alanine transaminase (ALT) activity levels in serum were measured (*n* = 6, ***P* < 0.01, two-way ANOVA, mean ± SEM). Abbreviations: AB, apoptotic body; ALT, alanine aminotransferase; ANOVA, analysis of variance; iAB, immunogenic apoptotic body; LPS, lipopolysaccharide; MPLA, monophosphoryl lipid A; PBS, phosphate-buffered saline; SEM, standard error of the mean.

**
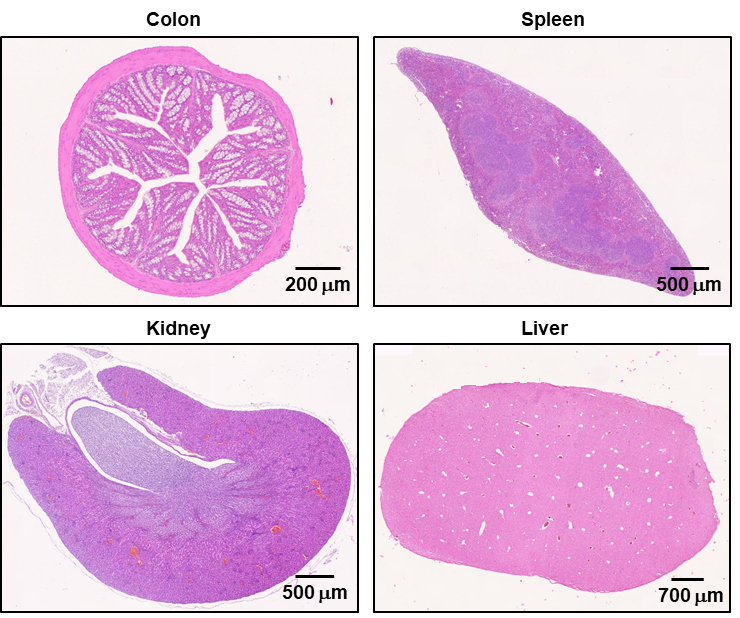
**

**Supplementary Figure S15.** **H&E staining of peripheral tissue**. BALB/c mice were administered with iAB-CT26 at 2-day intervals starting 7 days after tumor implantation. At 24 h after the final injection, peripheral tissues were harvested and stained with H&E. Representative images from six mice per condition are shown (*n* = 6 mice). Abbreviations: H&E, Hematoxylin & Eosin; iAB, immunogenic apoptotic body.
